# Glyoxal damages human aortic endothelial cells by perturbing the glutathione, mitochondrial membrane potential, and mitogen-activated protein kinase pathways

**DOI:** 10.1186/s12872-021-02418-3

**Published:** 2021-12-18

**Authors:** Ming-Zhang Xie, Chun Guo, Jia-Qi Dong, Jie Zhang, Ke-Tao Sun, Guang-Jian Lu, Lei Wang, De-Ying Bo, Lu-Yang Jiao, Guo-An Zhao

**Affiliations:** 1 Department of Laboratory, First Affiliated Hospital of Xinxiang Medical University, Xinxiang, 453000 Henan People’s Republic of China; 2 Henan Key Laboratory of Neural Regeneration (Henan Joint International Research Laboratory of Neurorestoratology for Senile Dementia), First Affiliated Hospital of Xinxiang Medical University, Xinxiang, 453000 Henan People’s Republic of China; 3 Department of Cardiovascular, First Affiliated Hospital of Xinxiang Medical University, Xinxiang, 453000 Henan People’s Republic of China; 4 Department of Integrating Western and Chinese of Internal Medicine, First Affiliated Hospital of Xinxiang Medical University, Xinxiang, 453000 Henan People’s Republic of China; 5 Department of Laboratory, Zibo Central Hospital, Zibo, 255036 Shandong People’s Republic of China

**Keywords:** Cardiovascular diseases, Human aortic endothelial cells, Mitogen-activated protein kinase pathways, Glyoxal, Laboratory indexes

## Abstract

**Background:**

Exposure to glyoxal, the smallest dialdehyde, is associated with several diseases; humans are routinely exposed to glyoxal because of its ubiquitous presence in foods and the environment. The aim of this study was to examine the damage caused by glyoxal in human aortic endothelial cells.

**Methods:**

Cell survival assays and quantitative fluorescence assays were performed to measure DNA damage; oxidative stress was detected by colorimetric assays and quantitative fluorescence, and the mitogen-activated protein kinase pathways were assessed using western blotting.

**Results:**

Exposure to glyoxal was found to be linked to abnormal glutathione activity, the collapse of mitochondrial membrane potential, and the activation of mitogen-activated protein kinase pathways. However, DNA damage and thioredoxin oxidation were not induced by dialdehydes.

**Conclusions:**

Intracellular glutathione, members of the mitogen-activated protein kinase pathways, and the mitochondrial membrane potential are all critical targets of glyoxal. These findings provide novel insights into the molecular mechanisms perturbed by glyoxal, and may facilitate the development of new therapeutics and diagnostic markers for cardiovascular diseases.

**Supplementary Information:**

The online version contains supplementary material available at 10.1186/s12872-021-02418-3.

## Introduction

Glyoxal (GX), the smallest dialdehyde, is the most widely used retarding agent in the synthesis of chemicals, such as novel bio-adhesives for wood [1–3], and is also found in the environment [4]. In humans, a high dietary glycemic load leading to insulin resistance causes alterations in glucose and lipid metabolism, and results in the production of excess aldehydes, such as GX and methylglyoxal, which are associated with the progression of certain diseases, including cardiovascular disorders [5, 6]. With respect to tissue dysfunction, GX has been implicated in the progression of several degenerative conditions, such as Alzheimer’s disease and diabetes mellitus, in which increased serotonin release from the cells stimulate serotonin-mediated intestinal motility [7]. Furthermore, the consumption of high-carbohydrate diets might also induce the endogenous formation of GX [6]. Regarding DNA damage and tumor growth, DNA duplexes can undergo intra- and inter-cross-linking damage through the formation of GX–guanine adducts [8, 9]. Exposure to 0.1% GX was found to cause a significant increase in tumor size in the small intestines of male and female mice [10]. GX can also trigger changes to erythrocytes; specifically, it induces the modification of arginine residues of HbA0-forming G-H1, which decreases free iron-mediated oxidative reactions. GX-derived G-H1-mediated changes in the structural and functional properties of the heme protein, observed in vivo, may be of clinical significance [11]. Erythrocytes take up exogenous GX and convert it primarily to glycolate, and approximately 1% of this is converted to oxalate. This pathway of oxalate formation may be enhanced in diabetes and other diseases associated with increased oxidative stress [12]. Glutathione (GSH), a reactive cysteine residue containing a tripeptide, is the major soluble antioxidant molecule in cells due to its abundance in the cytosol, nucleus, and mitochondrion [13–16]. Thioredoxin-1 (Trx1) mainly acts by cleaving the disulfide bonds of oxidized proteins, thus affecting intracellular scavenging of oxidative stress. This action relies on cell survival, cell growth, and gene transcription [17]. Finally, the enzymes involved in glycolysis are heavily modified by GX, which can significantly inhibit the activity of GAPDH, which can in turn contribute to the pathological processes by impairing glycolytic processes [18].

The toxic effects of aldehydes include protein damage caused by the exposure of cells to endogenous and exogenous aldehydes, which leads to the formation of covalent adducts with proteins, leading to dysfunction. In addition, DNA damage, such as DNA double strand breaks (DSBs) [19], DNA–protein crosslinks (DPCs), or DNA interstrand crosslinks (ICLs) [20], occurs when a protein or DNA base in one strand undergoes further reactions with aldehyde–DNA base adducts in the opposite strand [21]. The FANC pathway, which includes the complementation group A (FANCA), is the major underlying mechanism involved in the repair of ICLs induced by aldehydes [19].

Previous studies have shown that aldehydes are independent risk factors for cardiovascular disease [17–19]. For example, GX is associated with critical targets in the human aortic endothelial cells (HAECs), which are implicated in several cardiovascular diseases. GX is also involved in the development of abnormal structures and functions of the arteries, such as atherosclerosis [5, 6]. The main objective of this study was to examine the damage-inducing effects of GX on HAECs and to provide novel insights into the molecular mechanisms that are perturbed by GX, which may, in turn, facilitate the development of new therapeutics and diagnostic markers for cardiovascular diseases.

## Materials and methods

### Chemicals

Buthionine sulfoximine (BSO), iodoacetic acid (IAA), iodoacetamide (IAM), auranofin (Auro), and carbonyl cyanide *m*-chlorophenylhydrazone (CCCP) were purchased from Solarbio (Beijing, China); CCCP and Auro were solubilized in dimethyl sulfoxide (DMSO) before use. GX (40%, v/v) was obtained from Xiya Chemical Industry (Jinan, China) and was dissolved in Milli-Q water (Millipore, Burlington, MA, USA) before use. HAECs were purchased from Bena Culture Collection Co. (Beijing, China). Fanconi anemia complementation group A (FANCA) siRNA and siRNA-mate kit were purchased from GenePharma Co., Ltd. (Shanghai, China). The GSH peroxidase assay kit was obtained from the Nanjing Jiancheng Bioengineering Institute (Nanjing, China). Trx1, phospho extracellular signal regulated kinase (P-ERK), phospho c-Jun N-terminal kinase (P-JNK), and phospho p38 kinase (P-P38) antibodies were purchased from Abcam (Cambridge, UK), and enhanced chemiluminescence (ECL) western blotting substrate was obtained from Biosharp (Hefei, China). The mitochondrial membrane potential kit was purchased from Abbkine (Wuhan, China) and fetal bovine serum was obtained from Tianhang (Zhejiang, China).

### siRNA knockdown of FANCA

siRNA-mediated knockdown of FANCA was achieved using the siRNA-mate kit following the manufacturer’s protocol. Briefly, 1 × 10^5^ HAECs were plated with 500 μL of Dulbecco’s modified Eagle’s medium (DMEM) supplemented with fetal bovine serum (FBS) but without antibiotics in each well of a 24 well-plate and incubated at 37 °C in a humidified chamber with a steady supply of 5% CO_2_. After 24 h of incubation, 30–50% confluent cells were transfected with siRNA targeting FANCA to inhibit its expression. FANCA siRNA (50.1 nmol/L) and siRNA-mate transfection reagent (6 μL) were gently mixed at 20–30 °C for 10 min in 600 μL of DMEM without FBS to form siRNA–liposome complexes, which were then added to each well containing cells. HAECs were incubated in this transfection media for 8 h. The HAECs were continued to be maintained in DMEM with 10% inactivated FBS at 37 °C in a humidified incubator with 5% CO_2_ for 48–96 h. After incubation, the lysates were harvested for western blot analyses.

### Cell culture and survival assays

HAECs were cultured as described previously. For the survival assays with GX, 12 h after plating of the cells, the medium was replaced with fresh medium containing a range of indicated concentrations of GX (0, 50, 100, 150, and 200 μM) every day, and the cells were incubated for 8 days to form colonies. For survival assays, BSO, which can specifically inhibit γ-glutamylcysteine synthetase, was used to deplete the intracellular GSH concentration. HAECs were incubated in culture medium with 1 mM BSO for up to 72 h. Auro, an inhibitor of thioredoxin reductase which specifically interferes with the reduction of oxidized Trx1, was applied at a concentration of 0–6 µM for 24 h, while HAEC membrane permeability to protons was augmented with CCCP treatment (0–800 µM for 2 h).

HAECs were rinsed twice with PBS after treatment; then, the cells were incubated in fresh medium for 8 days to form colonies. Only colonies with more than 50 cells were counted. Drug lethal dose (LD_90_) values were determined based on the GX dose that produced 90% cell damage, as confirmed from the survival curves.

### Measurement of GSH levels

To determine the critical targets of GX, we assessed the changes in the intracellular GSH level. HAECs were treated with GX (LD_90_). Untreated cells were used as the negative control group. Using a GSH detection kit, GSH was measured immediately after treatment.

The biochemical principle of this protocol is that dithionitrobenzoic acid reacts with sulfhydryl compounds resulting in a yellowish coloration of the compounds, a process which is catalyzed by GSH. Briefly, HAECs (1 × 10^6^) were suspended and lysed by ultrasound after treatment with GX (LD_90_, 0.12 mM, for 8 days) or BSO. The samples were mixed with GSH (1 mM) and reagent 1 and incubated at 37 °C for 5 min. Following this, a reaction mixture of reagents 2–5 was added to the samples and incubated at 37 °C for 15 min. The GSH levels were measured using an absorbance microplate reader (Molecular Devices, San Jose, CA, USA) at 420 nm.

### Measurement of the redox state of Trx1

The samples with reduced and oxidized cysteine thiol groups were treated with GX, and Trx1 proteins bearing different charges were separated by urea-polyacrylamide gel electrophoresis (PAGE) and selectively detected by western blotting [22].

#### Preparation of electrophoretic migration markers

All cysteine residues were reduced by incubating HAECs with 3.5 mM dithiothreitol (DTT) in urea buffer (8 M urea, 100 mM tris–HCl [pH 8.2], and 1 mM EDTA) for 30 min at 37 °C. Electrophoretic migration markers were treated with 15 mM IAA + 15 mM IAM for alkylation at 37 °C for 15 min.

#### Preparation of the Trx1 proteins with an unknown redox state

After treatment with GX (LD_90_, 0.12 mM, for 8 days) or Auro, the Trx1 proteins were alkylated with 200 μL of urea buffer (30 mM IAA) for 15 min at 37 °C. Two milliliters of acetone–1 N HCl (4 °C, 98:2 v/v) was used to precipitate proteins, and unreacted IAA was removed by microcentrifugation for 5 min at 11,000 g at 4 °C. The pellet was resuspended and washed in acetone–1 N HCl–H_2_O (4 °C, 98:2:10 v/v/v) thrice by microcentrifugation for 5 min at 11,000 g at 4 °C. TRX1 proteins were then reduced by incubating them with 200 μL of urea buffer containing 3.5 mM DTT for 30 min at 37 °C. Trx1 proteins were then mixed with 10 mM IAM for 15 min at 37 °C.

#### Detection of the redox state of Trx1

The treated Trx1 samples and the electrophoretic migration markers were analyzed with an 8 M urea–PAGE gel, using a 2.5% stacking gel and 12% separating gel run at a constant current of 5 mA for 3.5 h. Proteins were transferred to a polyvinylidene fluoride (PVDF) membrane (Darmstadt, Germany) after electrophoresis and incubated at 4 °C for 24 h in TBST (20 mM tris–HCl [pH 7.6], 140 mM NaCl, and 0.1% Tween). The membranes were probed with the relevant primary antibody (1:20,000, ab109385, Abcam) at 4 °C overnight, and rinsed thrice with TBST. They were then incubated with the enzyme-labeled goat anti-rabbit IgG (1:5000) at 37 °C for 2 h. The membrane was incubated with the ECL western blotting substrate after washing with TBST. Chemiluminescence was quantified on a ChemiDoc XRS + system (Bio-Rad, Hercules, CA, USA) and the results were determined using the ImageJ software (Version 1.47).

### Measurement of the mitochondrial membrane potential

To determine critical GX targets, we determined the effect of GX on mitochondrial function. HAECs were treated with GX (LD_90_, 0.12 mM, for 8 days) or without GX (control) and subsequently assessed using a mitochondrial membrane potential assay kit. The measurements were taken immediately after treatment. Briefly, cells were washed and seeded onto a 96-well plate with 100 μL buffer/well. After treatment with GX (LD_90_) or CCCP, a working solution of the carbocyanine dye JC-1 (100 μL/well) was added, followed by incubation in the dark at 37 °C for 10 min. Results were measured on a Hitachi F-2500 fluorescence spectrophotometer (Tokyo, Japan) at λ ex = 529 nm and λ em = 590 nm.

### Western blotting

HAECs were washed with PBS and harvested for treatment with the protein inhibitor (Hefei, China); 30 μg of proteins were subjected to separation on a 10% resolving sodium dodecyl sulfate–polyacrylamide gel electrophoresis (SDS-PAGE) gel. The separated proteins were then transferred onto PVDF membranes using a blotting instrument (Bio-Rad). The samples were incubated with the relevant primary antibody at 4 °C overnight, after which they were blocked with 5% bovine serum albumin (20 mM tris–HCl [pH 7.6], 140 mM NaCl, and 0.1% Tween) for 2 h at 37 °C. The steps thereafter were the same as those mentioned in “Detection of the redox state of Trx1” section after incubation with the primary antibody.

### Measurement of DPCs

HAECs were treated with GX (LD_90_, 0.12 mM, for 8 days) or without GX (control) to measure DPCs [23]. Briefly, cells were processed with sarkosyl, mixed with 9.3 g of CsCl (Wako, Japan), and sedimented at 500,000 *g* at 20 °C for 4 h (CsCl density gradient centrifugation) to isolate chromosomal DNA. Fluorescein isothiocyanate was dissolved in dimethylformamide to a final concentration of 0.1 mM to label DPCs. DPCs were measured with a Hitachi F-2500 fluorescence spectrophotometer (Hitachi, Japan) (λ ex = 490 nm, λ em = 520 nm).

### Measurement of DSBs

HAECs were irradiated with X-rays (marker), treated with GX (LD_90_, 0.12 mM, for 8 days) or without GX (control), after which DSBs [19] were detected by static-field gel electrophoresis. Cells (1 × 10^4^) were loaded on plug molds, and the plugs were slid into 0.6% SeaKem gold agarose gels; electrophoresis was performed for 36 h at a field strength of 0.6 V/cm in 0.5× TBE buffer to isolate DSBs. The samples were detected with a UV transilluminator and the images was captured using a digital camera (Bio-Rad). The band intensity was subsequently determined using the ImageJ software (Version 1.47).

### Statistical analysis

Data are presented as mean ± standard deviation of results from three to five repetitions. Statistical analysis was performed using the SPSS 22 package. The *t*-test was used to evaluate the statistical differences for paired data.

## Results

### DNA repair is not important in attenuating GX toxicity

**Fig. 1:**
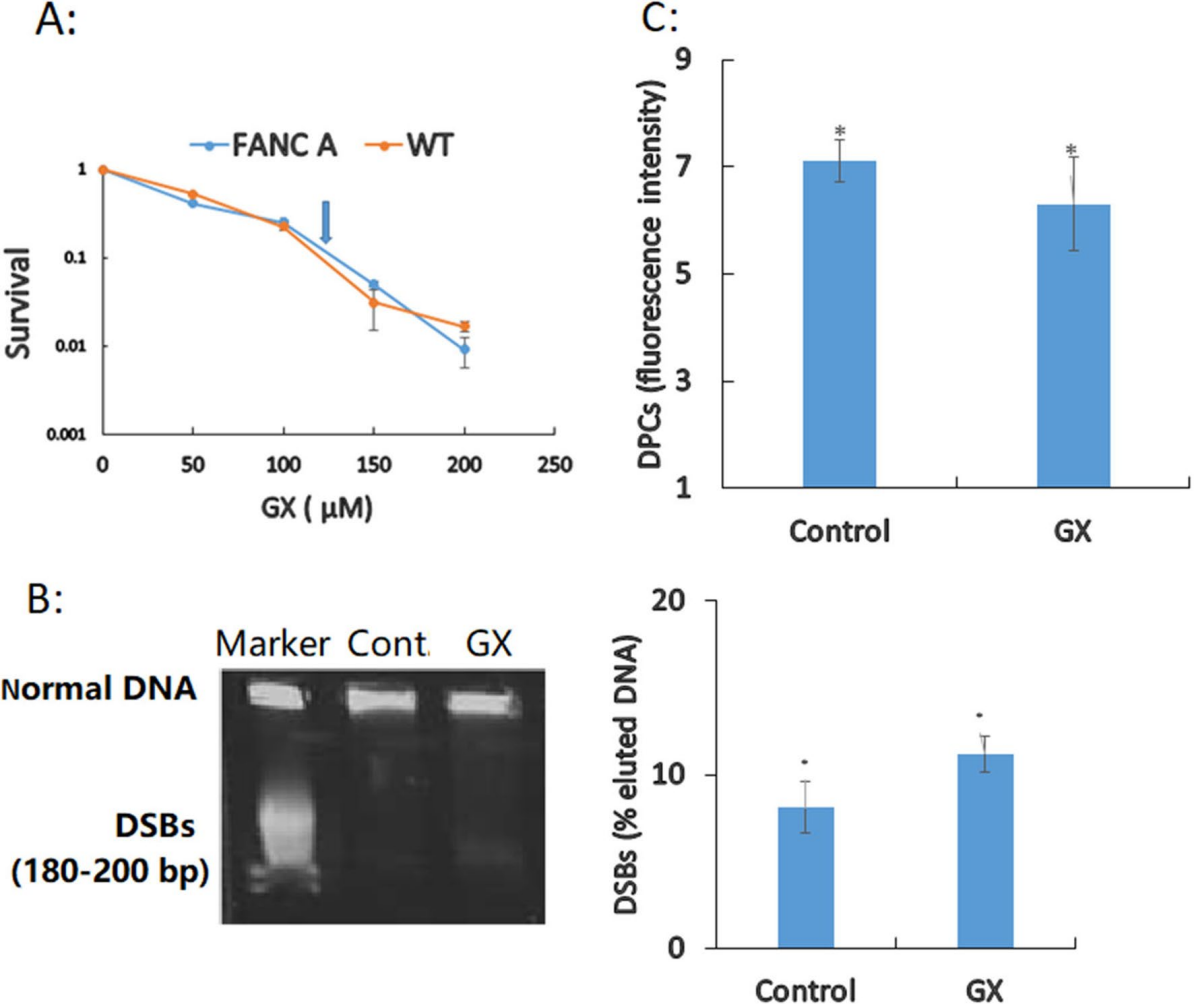
Analysis of DNA damage induced by glyoxal (GX) in human aortic endothelial cells (HAECs) **A** HAECs were treated with a range of concentrations (50, 100, 150, and 200 µM) of GX for 8 days, and their survival was determined (${\overline{\text{x}}}$ ± s, %). Drug lethal dose (LD_90_) values were confirmed from survival curves and are indicated by an arrow. **B** Double strand break (DSB) induction (uncropped blots/gels are presented in Additional file 1: Fig. S1). DSBs were detected using static-field gel electrophoresis after treatment with the fraction of DNA released relative to the total DNA. **C** DNA–protein crosslinks (DPC) induction. DPCs were quantified by the FITC-labelling method. Non-significant differences in the results obtained are denoted by n.s. (*p* > 0.05)

ICLs are amongst the most serious types of DNA damage induced by aldehydes, the accumulation of which results in growth arrest and cell death; the FANC pathway is capable of rescuing lCL-stalled replication forks while maintaining the genetic stability of the daughter cells to ensure survival [24, 25]. Therefore, a HAEC model deficient in the FANC pathway (Additional file 1: Fig. S4), as previously described [26], was used to determine whether GX generates ICLs in HAECs. The cells were exposed to GX in the culture medium to determine the tolerance mechanism to DNA damage. HAECs deficient in the FANC pathway (LD_90_ = 0.125 mM) were insensitive to GX compared to HAECs that were confirmed to be positive for the FANC pathway (LD_90_ = 0.12 mM) (Fig. 1A). Upon treating HAECs with GX (LD_90_, 0.12 mM, for 8 days), the DPC (Fig. 1C) and DSB (Fig. 1B) results were similar to those in the negative control group (*p* > 0.05). Thus, we concluded that DPCs, ICLs, and DSBs were not the primary reason for DNA damage (Fig. 1B, C).

### GX partially inactivates HAECs via disruption of GSH

**Fig. 2:**
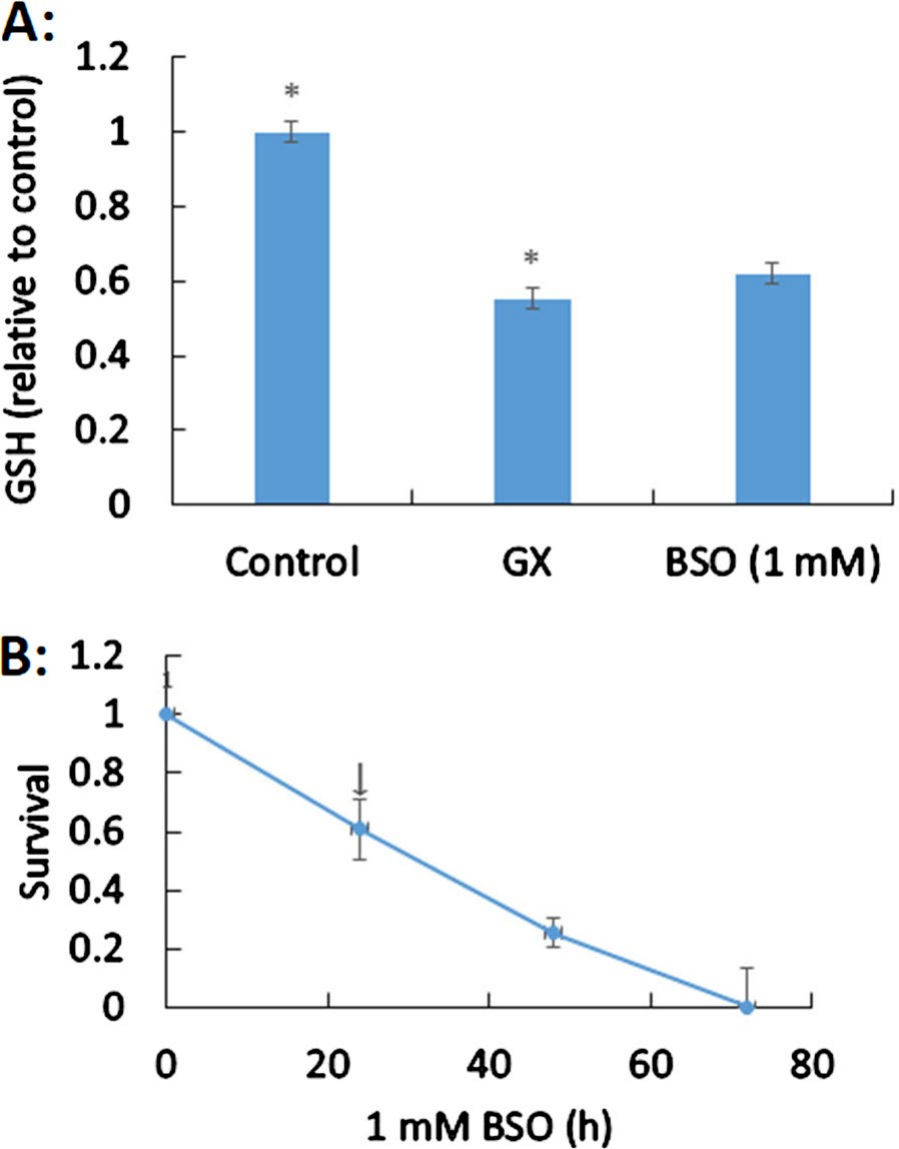
Effect of glyoxal (GX) on intracellular glutathione (GSH). **A** Data are presented as means ± standard deviations of three separate tests. Statistically significant differences are indicated by an asterisk, *p* < 0.05 indicates statistical significance. **B** Buthionine sulfoximine (BSO; 1 mM) was used to treat human aortic endothelial cells (HAECs) for different time periods, and cell survival was subsequently presented as means ± standard deviations

GSH is the thiol compound in cells which maintains the oxidoreductase balance and defends against oxidative damage. Pretreatment of HAECs with BSO decreased the intracellular GSH level (62% relative to untreated control cells). Intracellular GSH levels also decreased after treatment with GX (55% relative to the negative control group), as well as BSO (62% relative to untreated control cells; (Fig. 2A). BSO induced moderate damage (60% survival) as observed by evaluation with clonogenic assays (Fig. 2B). Thus, our results show that depletion of GSH occurs in the presence of GX, which contributes to cytotoxicity in HAECs.

### Depletion of mitochondrial membrane potential causes moderate cytotoxicity

**Fig. 3:**
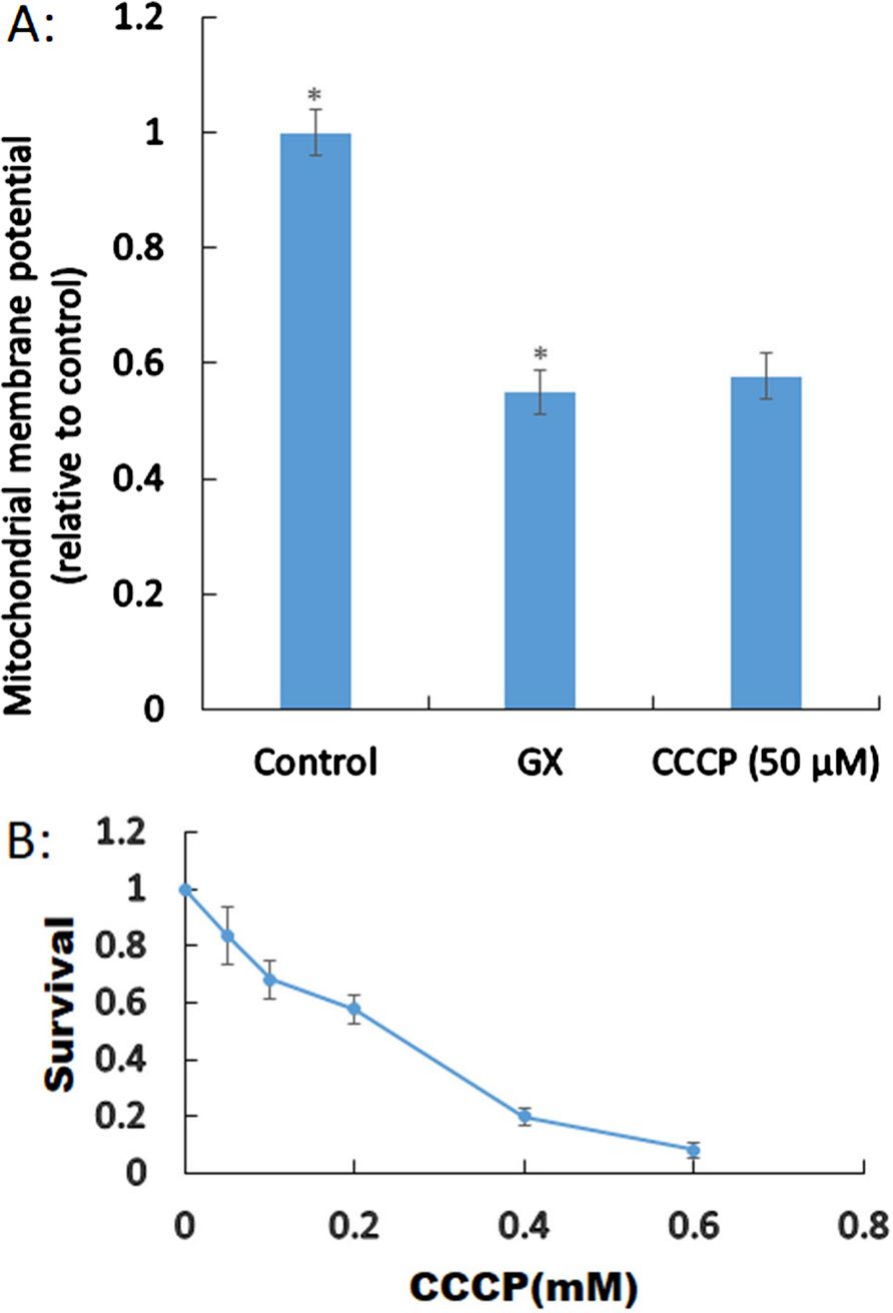
Effect of glyoxal (GX) on the mitochondrial membrane potential. **A** Human aortic endothelial cells (HAECs) were treated with GX. Statistically significant differences are indicated by an asterisk, *p* < 0.05 indicates statistical significance. **B** Survival of cyanide *m*-chlorophenylhydrazone (CCCP)-treated HAECs presented as means ± standard deviations

Monitoring alterations in the electric potential generated by the respiratory chain can indicate the functional metabolic status of mitochondria, and energy resistance inhibitors, such as CCCP, can be used to assess changes in mitochondrial membrane potential. After treatment with CCCP, the membrane permeability of HAECs was augmented with respect to protons; the cells showed decreased mitochondrial membrane potential (58% relative to that in negative control cells) compared to those treated with GX (55% relative to that in the untreated control cells; Fig. 3A). CCCP induced HAEC damage (84% survival), as observed by the clonogenic assay (Fig. 3B). Thus, GX-induced disruption of the mitochondrial membrane potential is an important reason for cell damage.

### Oxidation of Trx1 causes moderate cytotoxicity

**Fig. 4:**
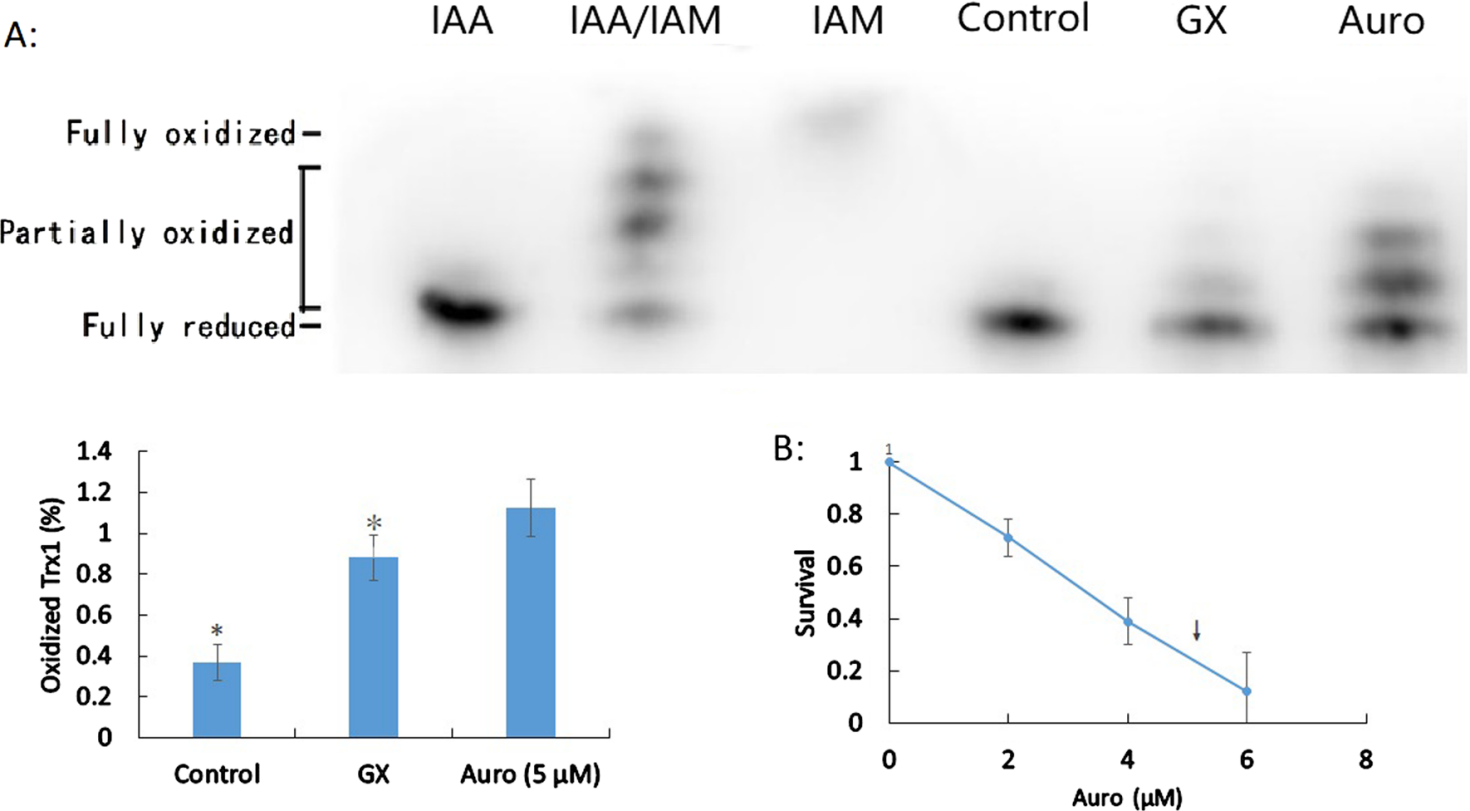
Redox state of Trx1. **A** Effect of glyoxal (GX) on the redox state of Trx1 (uncropped blots/gels are presented in Additional file 1: Fig. S2). Trx1 was either in a completely reduced state (iodoacetic acid (IAA) band) or in a completely oxidized state (iodoacetamide (IAM) band). Statistical differences are indicated by an asterisk, *p* < 0.05 indicates statistical significance. **B** Survival of auranofin-treated human aortic endothelial cells (HAECs) presented as means ± standard deviations

Trx1, an efficient substrate in the mammalian cytosol, expresses oxidoreductase activity to support the intracellular redox state, and Auro inhibits the activity of thioredoxin reductase (TrxR). To determine critical GX targets, we assessed the effect of GX on the oxidation of Trx1 and the resulting proteins. Several distinct protein bands were obtained by western blotting based on different Trx1 redox states (Fig. 4A). An upper shift of bands was observed immediately after treatment with GX (LD_90_; GX band = 1.87 relative to that in the untreated control cells; Fig. 4A). Treatment with Auro, an inhibitor of thioredoxin reductase (TrxR) which interferes with the reduction of oxidized Trx1, resulted in an upper shift of bands (Auro band = 3.29 relative to that in untreated control cells). Thus, the rates of oxidized Trx1 with GX treatment were altered compared to those in the untreated control cells. The results showed a dramatic decrease in cell survival (32% survival) after the treatment with Auro when analyzed by the clonogenic assay (Fig. 4B). Thus, oxidized Trx1, which damages the activities of peroxiredoxins and the cleaving of the disulfide bonds of oxidized proteins, was subjected to interference by GX and showed moderate cytotoxicity.

### Activation of mitogen-activated protein (MAP) kinase pathways, including P-ERK, P-JNK, and P-P38

**Fig. 5:**
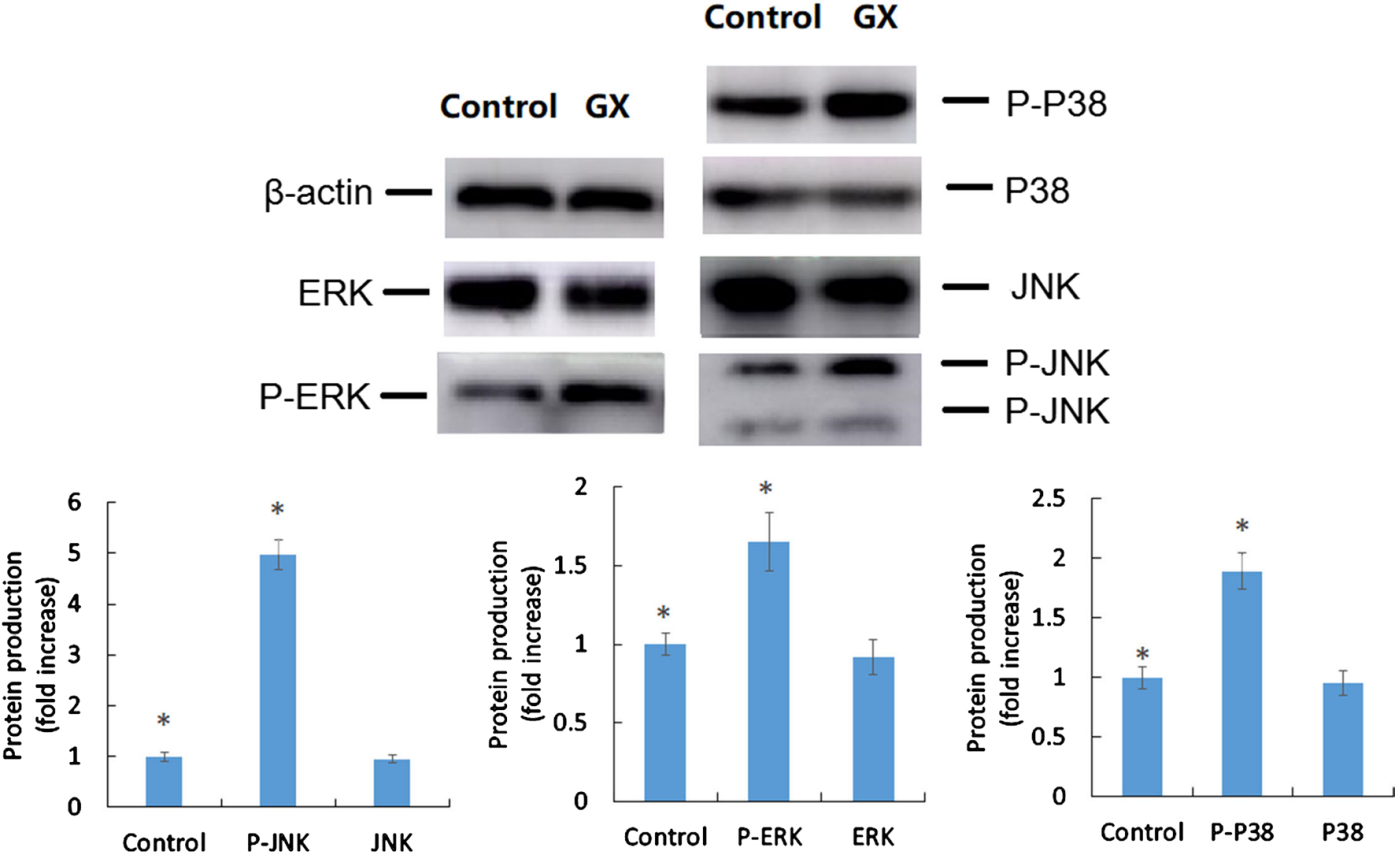
Effect of glyoxal (GX) on MAP kinase pathways (uncropped blots/gels are presented in Additional file 1: Fig. S3). Human aortic endothelial cells (HAECs) were treated with or without GX, lysed, and subjected to immunoblot assay with anti-phosphorylation or non-phosphorylation- level antibodies of MAP kinase pathways. Statistically significant differences are indicated by an asterisk, *p* < 0.05 indicates statistical significance

MAP kinase pathways include the phosphorylated (P-ERK, P-JNK, and P-P38) and non-phosphorylated (ERK, JNK, and P38) states which integrate and transmit signals for cell growth, differentiation, inflammatory responses, and apoptosis. To determine the effect of GX on the phosphorylated and non-phosphorylated states of the MAP kinase pathways, HAECs were treated with GX (LD_90_) and without GX (control) and the effects were measured immediately. Results showed that the levels of the non-phosphorylated states were not significantly different (*p* > 0.05, Fig. 5A). The expression levels of the phosphorylated MAP kinase pathway members increased immediately after treatment with GX, including P-ERK (105%), P-JNK (314%), and P-P38 (159%) (*p* < 0.05, Fig. 5B), compared to those in the negative control group.

## Discussion

As an important pollutant, endogenous GX can be produced by the metabolism of carbohydrates and oxidation of lipids or nucleic acids, whereas exogenous GX is present in foods and is released into the environment through human activities such as smoking [27, 28]. Aldehydes differ in their reactivity and damaging effects in HAECs due to their distinct chemical structures [24, 29]. However, to our knowledge, no study has identified the targets of critical damage in HAECs or the mechanism of protein damage resulting from GX cytotoxicity in conjunction with that of DNA damage, since concurrent DNA damage by dialdehydes yields similar results [24, 30].

ICLs constitute the most serious types of DNA damage induced by aldehydes. The primary repair mechanism of ICL damage induced by aldehydes is the FANC pathway [24, 25]. Previous studies have shown that ICLs, but not DPCs and DSBs, cause the critical cytotoxicity mediated by monoaldehydes [24, 25]. This study explored the effects of DNA damage-induced cytotoxicity by a dialdehyde (GX). We found that the FANC repair-deficient pathway is not essential for cell survival by mitigating the cytotoxicity of GX. Additionally, the DPCs, ICLs, and DSBs were not the primary reasons underlying cytotoxicity. A previous study had confirmed that ICLs induced by GX in human leukocytes were correlated with type 2 diabetes mellitus [8]. The differences in results from those of HAECs could be attributed to the use of different cell types. Our results differ from the those of previous studies on monoaldehydes as dialdehydes and monoaldehydes are completely different in their chemical structures [24, 31]. To the best of our knowledge, this is the first study to report a relationship between DNA damage and the pathologically significant induction of cardiovascular disease by dialdehydes.

GSH in an important antioxidant that has medical applications for anti-decrepitude and in liver protection, free radical scavenging, cancer treatment, and incretion maladjustment in clinic [13–15]. Our results showed that the major cytotoxic mechanism by which GX induced cardiovascular disease includes the depletion of intracellular GSH and the collapse of mitochondrial membrane potential. Previous research has shown that GX induces oxidative stress in various diseases, such as diabetic mellitus [32]. Our results are also similar to those of previous studies on monoaldehydes [24, 30, 32]. Previous studies showed that GX suppressed the activity of antioxidant enzymes such as super oxide dismutase (SOD) and induced an increase in the level of reactive oxygen species thereby promoting lipid peroxidation. Gallic acid can protect against GX-induced renal fibrosis [33]; Another research showed that the activities of both SOD and catalase were not significantly different after treatment with GX, though they were significantly increased by melthyglyoxal in human skin fibroblasts. They also reported that GX suppressed glutathione reductase and decreased the reduction of glutathione, which are similar to the results obtained in this study [34].

Endogenous antioxidant pathways, such as Trx1, protect against increased oxidative stress. Trx1 has been reported as a cornerstone in maintaining the cellular redox status and regulating signaling mechanisms [17, 35]. We also identified the role of oxidized Trx1 in inducing cardiovascular disease mediated by GX by using a modified western blot analysis. GX-mediated reduction of oxidized Trx1 resulted in partial HAEC cytotoxicity. To the best of our knowledge, this is the first report to directly assess the reduction of oxidized Trx1 in HAECs using the modified western blot analysis. Previous studies have indicated similar results which showed that dialdehydes (methylglyoxal) can assist glyoxalase and Trx1 at low concentrations but have an opposite effect at high concentrations [36]. Chemical modification of Trx1 by monoaldehydes can contribute to the development of cardiovascular disease by interfering with the redox signaling functions of Trx1 [24, 36]. However, the underlying reason needs to be further elucidated.

We also determined the effect of GX on the activation of MAP kinase pathways using western blot analysis and determined that they indeed contain critical targets, including P-ERK, P-JNK, and P-P38 MAPK, in HAECs. Previous studies have shown that P-JNK and P-ERK induce human cell apoptosis [37, 38]. To the best of our knowledge, this is the first study to directly assess the relationship between HAEC apoptosis and the activation of MAP kinase pathways induced by GX.

In short, the critical effects of GX include the depletion of intracellular GSH, activation of MAP kinase pathways, and collapse of the mitochondrial membrane potential. Oxidative stress induces the depletion of intracellular GSH, and suppression of the p38 MAPK signaling pathway can alleviate oxidative stress [39]. P-P38 MAPK can induce oxidative stress and apoptosis in human lens epithelial cells [40]. Thus, previous studies have shown that affecting these critical targets can damage HAECs.

## Conclusions

GX is a serious pollutant that humans are extensively exposed to, and subsequently, it is a major underlying factor in cardiovascular disorders. However, thus far, the molecular mechanisms underlying this action of GX are unknown. This study showed that the critical targets of GX include the depletion of intracellular GSH, the activation of MAP kinase pathways, and the collapse of mitochondrial membrane potential; we also showed that DNA repair is not important in attenuating GX toxicity. The present findings provide novel insights into the understanding of these molecular mechanisms and may help in the development of new therapeutic targets and diagnostic markers in cardiovascular disease, as well as to provide a reference for the progression of precision medicine.

## Supplementary Information

**Additional file 1.** siRNA-mediated Knockdown of FANCA.

**Additional file 2.** Original, Unprocessed Versions of Double Srand Break (DSB) Induction.

**Additional file 3.** Original, Unprocessed Versions of Effect of Glyoxal (GX) on the Redox State of Trx1.

**Additional file 4.** Original, Unprocessed Versions of Effect of Glyoxal (GX) on MAP Kinase Pathways.

## Data Availability

The dataset(s) supporting the conclusions of this article is(are) included within the article (and its additional file(s)).

## Abbreviations

GX: Glyoxal
GSH: Glutathione
Trx1: Thioredoxin-1
DSBs: DNA double strand breaks
DPCs: DNA–protein crosslinks
ICLs: DNA interstrand crosslinks
HAECs: Human aortic endothelial cells
BSO: Buthionine sulfoximine
IAA: Iodoacetic acid
IAM: Iodoacetamide
Auro: Auranofin
CCCP: Carbonyl cyanide *m*-chlorophenylhydrazone
DMSO: Dimethyl sulfoxide
P-ERK: Phospho extracellular signal regulated kinase
P-JNK: Phospho c-Jun N-terminal kinase
P-P38: Phospho p38 kinase
ECL: Enhanced chemiluminescence
FBS: Fetal bovine serum
LD_90_: Drug lethal dose
DTT: Dithiothreitol
PVDF: Polyvinylidene fluoride membrane
SDS–PAGE: Sodium dodecyl sulfate–polyacrylamide gel electrophoresis
TrxR: Thioredoxin reductase
MAP: Mitogen-activated protein
SOD: Super oxide dismutase
