# Supplementary material for: Glyoxal damages human aortic endothelial cells by perturbing the glutathione, mitochondrial membrane potential, and mitogen-activated protein kinase pathways
Source: BMC Cardiovasc Disord. 2021 Dec 18;21:603. doi: 10.1186/s12872-021-02418-3 (PMC8684178; doi:10.1186/s12872-021-02418-3)
Supplement: Supplementary file 1 — Additional file 1. siRNA-mediated Knockdown of FANCA. [file 12872_2021_2418_MOESM1_ESM.docx]

Supplementary materials


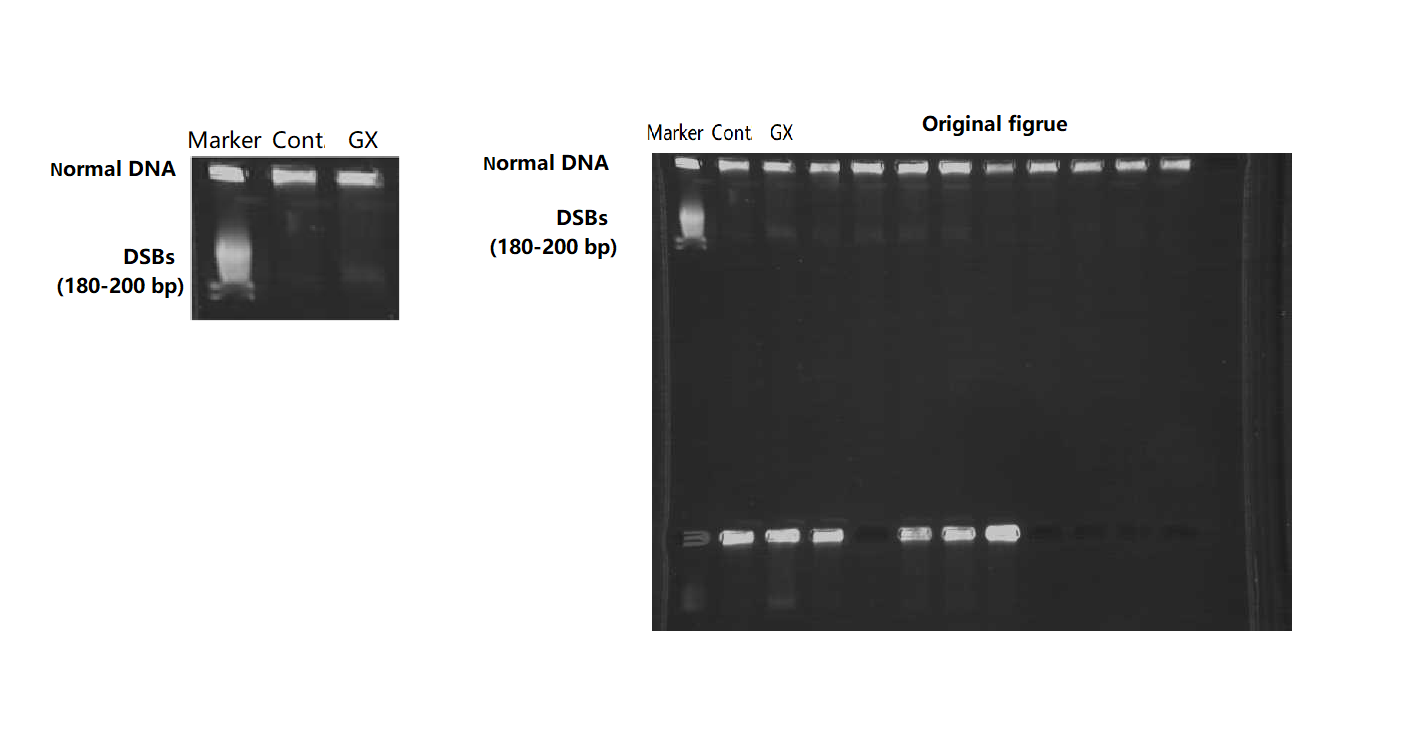


Supplementary Fig 1. the Original, Unprocessed Versions of Double Srand Break (DSB) Induction
