# Supplementary material for: Glyoxal damages human aortic endothelial cells by perturbing the glutathione, mitochondrial membrane potential, and mitogen-activated protein kinase pathways
Source: BMC Cardiovasc Disord. 2021 Dec 18;21:603. doi: 10.1186/s12872-021-02418-3 (PMC8684178; doi:10.1186/s12872-021-02418-3)
Supplement: Supplementary file 4 — Additional file 4. Original, Unprocessed Versions of Effect of Glyoxal (GX) on MAP Kinase Pathways. [file 12872_2021_2418_MOESM4_ESM.docx]

Supplementary materials


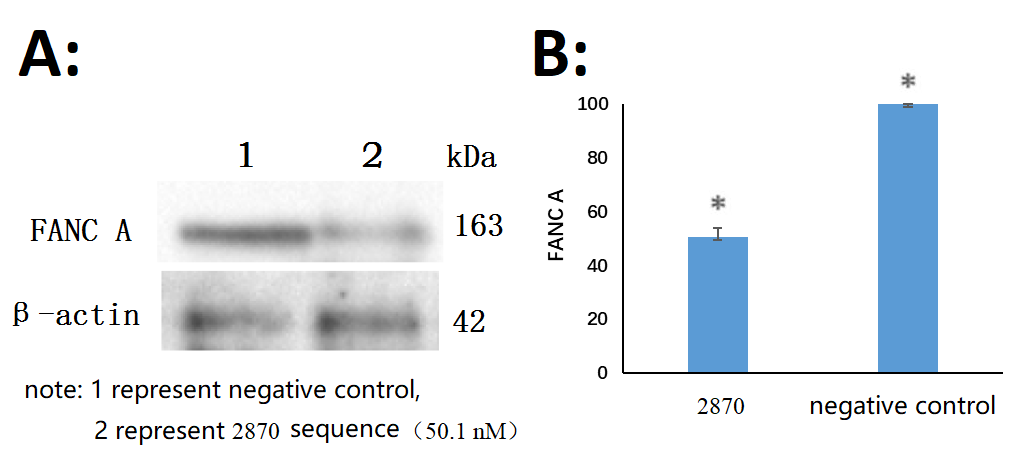


Supplementary Fig. 4 siRNA-mediated Knockdown of FANCA.

Supplementary Fig. 4 siRNA-mediated knockdown of FANCA. (A) Immunoblot assay of Human aortic endothelial cells (HAECs) with anti-FANCA antibody. Lane 1 contains the cell lysate prepared from HAECs transfected with the negative control (scrambled siRNA). Lane 2 has the lysate of HAECs deficient in the FANC pathway. β-actin is used as a loading control (B) Quantitative estimation of siRNA-mediated knockdown of FANCA. Statistically significant differences are indicated by the asterisk. p < 0.05 indicates statistical significance.
